# White matter microstructure in late middle-age: Effects of apolipoprotein E4 and parental family history of Alzheimer's disease

**DOI:** 10.1016/j.nicl.2014.04.008

**Published:** 2014-04-21

**Authors:** Nagesh Adluru, Daniel J. Destiche, Sharon Yuan-Fu Lu, Samuel T. Doran, Alex C. Birdsill, Kelsey E. Melah, Ozioma C. Okonkwo, Andrew L. Alexander, N. Maritza Dowling, Sterling C. Johnson, Mark A. Sager, Barbara B. Bendlin

**Affiliations:** a Waisman Laboratory for Brain Imaging and Behavior, Madison, WI, USA; b Wisconsin Alzheimer's Disease Research Center, University of Wisconsin School of Medicine and Public Health, Department of Medicine, 600 Highland Avenue, Madison, WI 53792, USA; c Department of Medical Physics, University of Wisconsin School of Medicine and Public Health, 1111 Highland Ave, Madison, WI 53705, USA; d University of Wisconsin School of Medicine and Public Health, Department of Psychiatry, 6001 Research Park Blvd, Madison, WI 53719, USA; e Department of Biostatistics and Medical Informatics, 600 Highland Avenue, Madison, WI 53792, USA; f Geriatric Research, Education and Clinical Center (GRECC), William S. Middleton Memorial Veteran's Hospital, 2500 Overlook Terrace, Madison, WI 53705, USA; g Wisconsin Alzheimer's Institute, University of Wisconsin School of Medicine and Public Health, 7818 Big Sky Drive, Madison, WI 53719, USA

**Keywords:** Alzheimer's disease, family history, APOE4, diffusion tensor imaging, MRI, risk factors, age, sex

## Abstract

**Introduction:**

Little is still known about the effects of risk factors for Alzheimer's disease (AD) on white matter microstructure in cognitively healthy adults. The purpose of this cross-sectional study was to assess the effect of two well-known risk factors for AD, parental family history and APOE4 genotype.

**Methods:**

This study included 343 participants from the Wisconsin Registry for Alzheimer's Prevention, who underwent diffusion tensor imaging (DTI). A region of interest analysis was performed on fractional anisotropy maps, in addition to mean, radial, and axial diffusivity maps, aligned to a common template space using a diffeomorphic, tensor-based registration method. The analysis focused on brain regions known to be affected in AD including the corpus callosum, superior longitudinal fasciculus, fornix, cingulum, and uncinate fasciculus. Analyses assessed the impact of APOE4, parental family history of AD, age, and sex on white matter microstructure in late middle-aged participants (aged 47–76 years).

**Results:**

Both APOE4 and parental family history were associated with microstructural white matter differences. Participants with parental family history of AD had higher FA in the genu of the corpus callosum and the superior longitudinal fasciculus. We observed an interaction between family history and APOE4, where participants who were family history positive but APOE4 negative had lower axial diffusivity in the uncinate fasciculus, and participants who were both family history positive and APOE4 positive had higher axial diffusivity in this region. We also observed an interaction between APOE4 and age, whereby older participants (=65 years of age) who were APOE4 carriers, had higher MD in the superior longitudinal fasciculus and in the portion of the cingulum bundle running adjacent to the cingulate cortex, compared to non-carriers. Older participants who were APOE4 carriers also showed higher radial diffusivity in the genu compared to non-carriers. Across all participants, age had an effect on FA, MD, and axial and radial diffusivities. Sex differences were observed in FA and radial diffusivity.

**Conclusion:**

APOE4 genotype, parental family history of AD, age, and sex are all associated with microstructural white matter differences in late middle-aged adults. In participants at risk for AD, alterations in diffusion characteristics—both expected and unexpected—may represent cellular changes occurring at the earliest disease stages, but further work is needed. Higher mean, radial, and axial diffusivities were observed in participants who are more likely to be experiencing later stage preclinical pathology, including participants who were both older and carried APOE4, or who were positive for both APOE4 and parental family history of AD.

## Highlights

• Focused on participants (*N* = 343) in the Wisconsin Registry for Alzheimer's Prevention
• Examined effects of Alzheimer's disease risk on brain white matter utilizing DTI
• Parental family history was unexpectedly associated with higher fractional anisotropy.
• Lower diffusivities in certain risk groups may indicate early disease pathology.
• Results have implications for understanding preclinical disease pathology.

## 1 Introduction

Axonal degeneration and myelin degeneration are known features of Alzheimer's disease (AD) (Bronge et al., 2002; Chalmers et al., 2005; de la Monte, 1989; Ihara et al., 2010; Roher et al., 2002; Scheltens et al., 1995). Brain imaging studies show reduced white matter volume in AD (Balthazar et al., 2009; Baxter et al., 2006; Chaim et al., 2007; Im et al., 2008; Li et al., 2008; Salat et al., 2009; Stout et al., 1996; Teipel et al., 1998, 2002; Vermersch et al., 1994; Wang et al., 2006), white matter atrophy over time (Hua et al., 2010) and microstructural alterations as indexed by diffusion tensor imaging (DTI) (Bozzali et al., 2002; Canu et al., 2009; Duan et al., 2006; Fellgiebel et al., 2008; Hanyu et al., 1999; Huang et al., 2007, 2012; Medina et al., 2006; Rose et al., 2008; Salat et al., 2010; Stahl et al., 2007; Takahashi et al., 2002; Wang et al., 2012; Xie et al., 2006). Brain changes in AD occur several years before memory symptoms appear, and accordingly, risk factors for AD are also associated with white matter changes, including apolipoprotein E e4 genotype (APOE4) (Bartzokis et al., 2006; Bartzokis et al., 2007; Filippini et al., 2009; Heise et al., 2011; Honea et al., 2009; Nierenberg et al., 2005; Persson et al., 2006; Ryan et al., 2011; Westlye et al., 2012) and parental family history of AD (Bendlin et al., 2010; Xiong et al., 2011).

While abnormalities in diagnosed disease are becoming well-known, the number of studies that clarify the pattern of early AD-related white matter change in at-risk populations is still low. Particularly lacking are studies of white matter health and AD risk that focus on middle-age. Given that AD pathogenesis may be related to patterns of white matter development over the lifespan (Bartzokis, 2004), the purpose of this study was to map out the effect of AD risk factors on white matter microstructure in late middle-age, a stage when AD-related brain degeneration is suspected to be already occurring (Bendlin et al., 2010; Chen et al., 2007, 2012; Fleisher et al., 2005; Johnson et al., 2007; Langbaum et al., 2012; Okonkwo et al., 2012; Patel et al., 2013; Reiman et al., 1996, 2001; Xiong et al., 2011; Xu et al., 2009b).

Participants were recruited from the Wisconsin Registry for Alzheimer's Prevention, a well-characterized cohort of adults who are followed longitudinally (Sager et al., 2005). We utilized DTI, a highly sensitive method for characterizing microstructural differences in the brain. The study examined fractional anisotropy (FA) and mean diffusivity (MD), in addition to axial diffusivity and radial diffusivity. FA is a sensitive scalar measure of the directional coherence of water diffusion that reflects tissue microstructure and is especially sensitive to white matter features that include myelination, axonal diameter, axonal density and cytoskeletal features (Alexander et al., 2007; Beaulieu, 2002). MD is the average of the three diffusion tensor eigenvalues and is inversely modulated by the density of tissue membranes and is sensitive to cellular structure, edema, and necrosis (Alexander et al., 2011). Axial and radial diffusivities may provide additional information, as several animal studies suggest that they are related to axonal and myelin health, respectively (Bennett et al., 2012; Budde et al., 2007; Feng et al., 2009; Harsan et al., 2006; Hofling et al., 2009; Janve et al., 2013; Song et al., 2002, 2005; Sun et al., 2006; Wu et al., 2007).

In order to limit the number of comparisons, we focused on select regions of interest chosen based on their vulnerability to AD. In particular, we were interested in examining white matter tracts that subserve gray matter regions affected by AD. The medial and lateral parietal, medial and lateral temporal, and inferior frontal cortices show substantial structural and functional changes in AD (Lee et al., 2003; Poulin and Zakzanis, 2002; Reiman and Jagust, 2012), and accordingly, the fiber tracts connecting these cortical regions have also shown alteration in AD, including the uncinate (Kiuchi et al., 2009; Villain et al., 2010; Yasmin et al., 2008; Zhang et al., 2009), fornix (Huang et al., 2012; Mielke et al., 2009; Oishi et al., 2012), cingulum (Canu et al., 2013; Liu et al., 2011; Villain et al., 2008; Zhang et al., 2007c, 2009), superior longitudinal fasciculi (Benitez et al., 2013; Liu et al., 2011), and the corpus callosum (Di Paola et al., 2010a; Pitel et al., 2010; Teipel et al., 2002; Teipel et al., 2012). We hypothesized that participants with risk factors for AD would show lower FA, higher MD, higher radial diffusivity and higher axial diffusivity in these white matter regions compared to participants without risk. While risk for Alzheimer's was our primary focus, the study provided an opportunity to examine the effects of age and sex in a large sample. Based on prior studies—including work from our group—suggesting that older age, sex, and carrying multiple risk factors contribute to the degree of presymptomatic AD-related neural damage, we also tested for interactions between age and risk (APOE4 or parental family history) (Ryan et al., 2011), sex and risk (Damoiseaux et al., 2012), and APOE4 and parental family history (Bendlin et al., 2010).

## 2 Materials and methods

Study procedures were approved by the University of Wisconsin Health Sciences Institutional Review Board and were in accordance with U.S. federal regulations. All participants provided written informed consent.

### 2.1 Participants

Three hundred fifty-eight participants from the Wisconsin Registry for Alzheimer's Prevention (WRAP) underwent brain imaging as part of studies on memory, aging, and risk for AD. WRAP is a registry of cognitively normal adults who are followed longitudinally and comprise a cohort whose members either have a family history (FH) of late onset AD or no family history of AD (Sager et al., 2005). A positive family history was defined as having one or both parents with autopsy-confirmed or probable AD as defined by research criteria (McKhann et al., 1984; McKhann et al., 2011), reviewed by a multidisciplinary diagnostic consensus panel. Absence of family history of AD was verified through detailed medical history surveys and phone interview with the participants. Further, absence of family history of AD required that the father survive to at least age 70 and the mother to age 75 without incurring a formal diagnosis of dementia or exhibiting cognitive deterioration.

The inclusion criteria for this study consisted of the following: normal cognitive function determined by neuropsychological evaluation, no contraindication for magnetic resonance imaging (MRI) and a subsequent normal MRI scan, no current diagnosis of major psychiatric disease or other major medical conditions (e.g., myocardial infarction, or recent history of cancer), and no history of head trauma. All participants underwent MRI and neuropsychological testing. Based on comprehensive cognitive testing, four cognitive factor scores were derived from a factor analytic study of the WRAP neuropsychological battery and adapted from work published in Dowling et al. (2010). Factor scores represented cognitive domains known to change with age: Immediate Memory, Verbal Learning and Memory, Working Memory, and Speed and Flexibility.

A total of 15 participants were excluded from the final analysis. Four were removed due to compromised data quality, and two participants were removed due to mild cognitive impairment as shown by cognitive testing. An additional nine participants were excluded due to brain abnormalities found by the reviewing radiologist. The final sample comprised 343 participants; demographics and mean cognitive factor scores are provided in Table 1.

### 2.2 APOE genotyping

Determination of APOE genotype has previously been described (Johnson et al., 2011). APOE alleles were classified as having an e2, e3, or e4 isoform. Participants were distinguished using a binary categorical variable, where participants were classified as carriers (one or more e4 alleles present) or non-carriers (no e4 allele present).

### 2.3 Magnetic resonance imaging

#### 2.3.1 DTI acquisition

Participants were imaged on a General Electric 3.0 Tesla Discovery MR750 (Waukesha, WI) MRI system with an 8-channel head coil and parallel imaging (ASSET). DTI was acquired using a diffusion-weighted, spin-echo, single-shot, echo planar imaging (EPI) pulse sequence in 40 encoding directions at *b* = 1300 s/mm^2, with eight non-diffusion weighted (*b* = 0) reference images. The cerebrum was covered using contiguous 2.5 mm thick axial slices, FOV = 24 cm, TR = 8000 ms, TE = 67.8 ms, matrix = 96 × 96, resulting in isotropic 2.5 mm^3^ voxels. High order shimming was performed prior to the DTI acquisition to optimize the homogeneity of the magnetic field across the brain and to minimize EPI distortions.

#### 2.3.2 Image analysis

The study was designed to take advantage of tools optimized for DTI analysis. When conducting analyses across a large sample, it is critical to ensure accurate registration of individual brain imaging maps into a common space. In order to ensure the best possible methods, we employed a robust processing pipeline, based on methods in Zhang et al. (2007a).

**Fig. 1:**
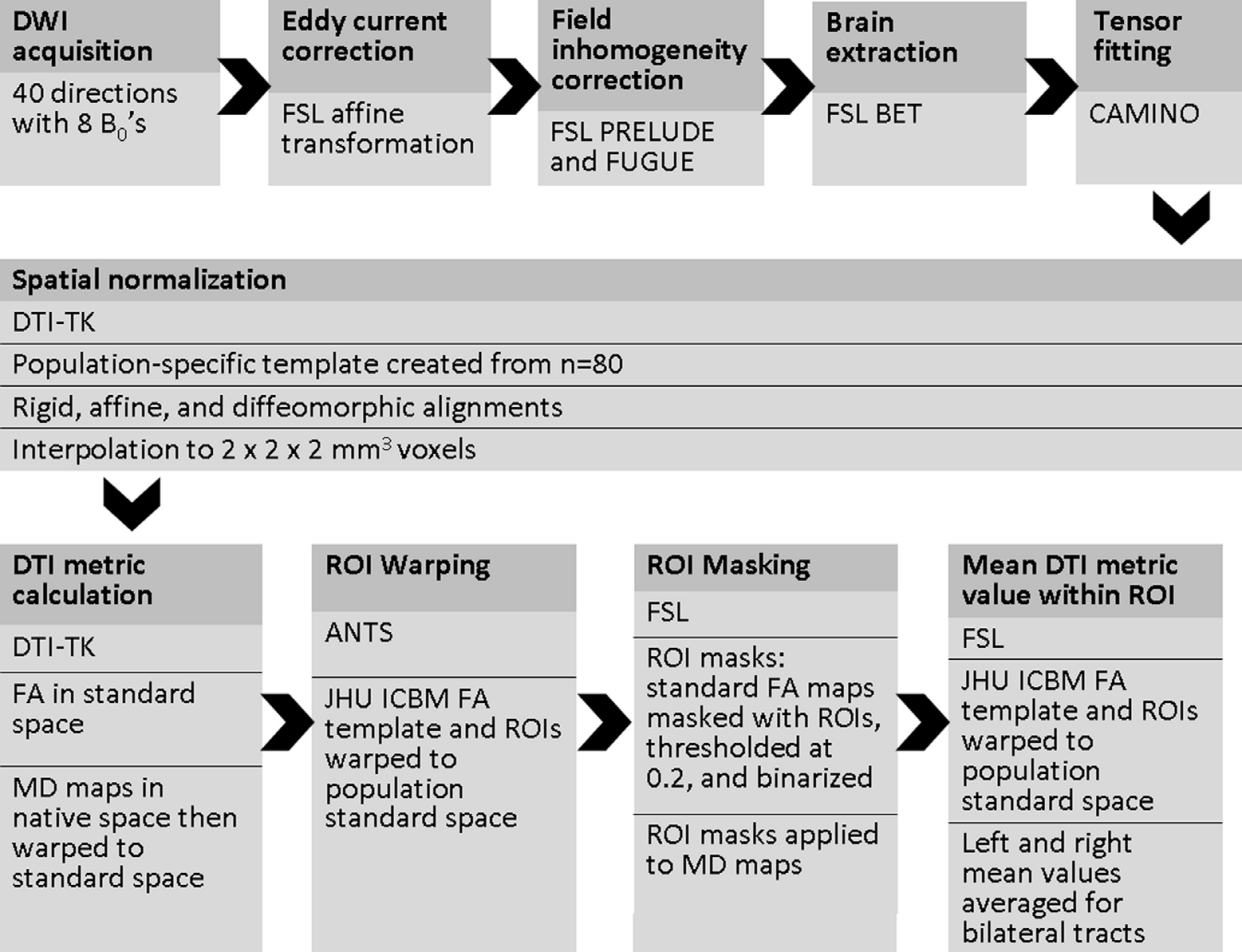
DTI processing stream. Shown here is the diffusion tensor imaging “processing pipeline”. Images underwent eddy current correction, field inhomogeneity correction, skull stripping and tensor fitting using tools from the FMRIB Software Library (FSL). Tensor fitting was performed using the University College London, Camino Diffusion MRI Toolkit. Next, images were normalized utilizing Diffusion Toolkit for DTI analysis (DTI-TK). Advanced Normalization Tools (ANTS) was used to warp the Johns Hopkins International Consortium for Brain Mapping (JHU ICBM) FA template and white matter regions of interest (ROIs) to the study's template space. ROIs were used to extract fractional anisotropy (FA), mean diffusivity (MD), axial diffusivity, and radial diffusivity values from individual subject DTI maps in template space. ROIs are depicted in Fig. 2.

The processing stream is depicted in Fig. 1. First, head motion and image distortions (stretches and shears) due to eddy currents were corrected with affine transformation in the FSL (FMRIB Software Library) package (http://www.fmrib.ox.ac.uk/fsl/). Geometric distortion from the inhomogeneous magnetic field applied was corrected with the *b* = 0 field map and PRELUDE (phase region expanding labeler for unwrapping discrete estimates) and FUGUE (FMRIB's utility for geometrically unwarping EPIs) from FSL. Twenty-nine of the participants that underwent diffusion-weighted imaging did not have field maps acquired during their imaging session. Because these participants did not differ in FH, APOE4 genotype, sex, or age compared to the participants that had field map correction, they were included in order to enhance the final sample size. All images are visually inspected at this stage to ensure that data with substantial artifact (loss of frontal or temporal lobe signal) or geometric distortions are not included in the final sample. Brain tissue was extracted using FSL's BET (Brain Extraction Tool). Tensor fitting was performed using a nonlinear least squares method in Camino (http://cmic.cs.ucl.ac.uk/camino/).

#### 2.3.3 Template creation

Individual maps were registered to a population specific template constructed using Diffusion Tensor Imaging ToolKit (DTI-TK), http://www.nitrc.org/projects/dtitk/, which is an optimized DTI spatial normalization and atlas construction tool (Wang et al., 2011; Zhang et al., 2006, 2007b) that has been shown to perform superior registration compared to scalar based registration methods (Adluru et al., 2012). The template is constructed in an unbiased way that captures both the average diffusion features (e.g., diffusivities and anisotropy) and the anatomical shape features (tract size) in the population (Zhang et al., 2007b). A subset of 80 diffusion tensor maps was used to create a common space template. All diffusion tensor maps were normalized to the template with rigid, affine, and diffeomorphic alignments and interpolated to 2 × 2 × 2 mm^3^ voxels. With DTI-TK, FA maps were calculated in the normalized space, while MD, ?1, ?2, and ?3 were calculated in native space then warped to the same normalized space as the FA maps with a 2 × 2 × 2 mm^3^ voxel dimension. All FA maps are visually inspected to rule out inclusion of maps with missing data in regions of interest or other artifacts.

#### 2.3.4 Registration

White matter alignment was performed using a diffeomorphic (topology preserving) registration method (Zhang et al., 2007a) that incrementally estimates the displacement field using a tensor-based registration formulation (Zhang et al., 2006). Tensor-based registration provides optimal alignment between subjects by taking advantage of similarity measures comparing whole tensors via explicit optimization of tensor reorientation (Alexander et al., 2001b; Alexander and Gee, 2000). By computing image similarity on the basis of full tensor images rather than scalar features, the algorithm incorporates local fiber orientations as features to drive the alignment of individual white matter tracts. Using full-tensor information is highly effective in spatially normalizing tract morphology and tensor orientation, and enhances sensitivity to microstructural variations (Zhang et al., 2007a).

**Fig. 2:**
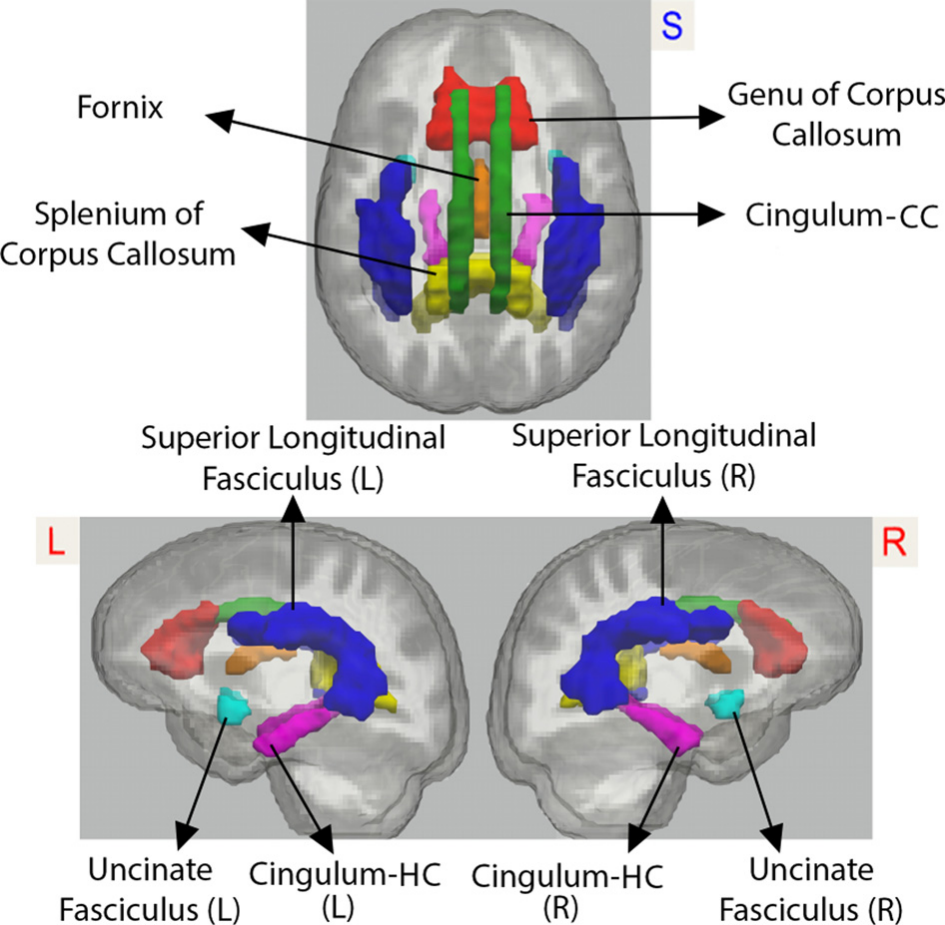
Regions of interest. We analyzed fractional anisotropy and mean, axial, and radial diffusivity values extracted from the regions of interest depicted here: genu of the corpus callosum (red), splenium of the corpus callosum (yellow), fornix (orange), superior longitudinal fasciculus, left and right combined (blue), cingulum bundle adjacent to the cingulate gyrus (called cingulum-CC in the manuscript text), left and right combined (green), cingulum projections to the hippocampus (called cingulum-HC in the manuscript text), left and right combined (magenta), and uncinate, left and right combined (blue). L = left, R = right.

The Johns Hopkins International Consortium for Brain Mapping (ICBM) FA template was warped to the study's template space using Advanced Normalization Tools (ANTS). ANTS has been demonstrated to be among the most accurate intensity-based normalization method among fourteen different methods (Klein et al., 2009). Using ANTS, the Johns Hopkins ROIs (Wakana et al., 2004) were individually warped to the study's template space. Seven ROIs were chosen for analysis, see Fig. 2: the genu and splenium of the corpus callosum, the fornix (body and column), the cingulum bundles adjacent to the cingulate cortex (cingulum-CC, left and right averaged), the cingulum bundle projections to the hippocampus (cingulum-HC, left and right averaged), the superior longitudinal fasciculi (SLF, left and right averaged), and the uncinate fasciculi (left and right averaged). The FA map for each subject in normalized template space was masked by the chosen ROIs. The resulting FA ROIs were thresholded at 0.2 to reduce inclusion of gray matter voxels in the white matter masks. The thresholded FA ROIs were binarized and used as ROI masks to isolate the white matter ROIs of each subject's standard space FA, MD, ?1 (axial diffusivity), and (?2 + ?3) / 2 (radial diffusivity) maps. Finally, the average values of FA, MD, and axial and radial diffusivities within each subject's ROI-masked maps were calculated.

### 2.4 Statistical analysis

#### 2.4.1 Brain image analysis

We conducted multivariate analyses of variance (MANOVAs) to test the effects of APOE4, FH, sex, and age, on FA, MD, and axial and radial diffusivities in all seven ROIs. In addition to main effects, we tested for all 2-way interactions, in addition to a 3-way interaction for  age * APOE4 * FH. The data set met the requirement of equal covariance matrices, as assessed with Box's M Test (*p* > 0.01). In all models, the independent factors of interest were APOE4, FH, age, and sex, while the dependent variables were either FA, MD, axial diffusivity, or radial diffusivity for all 7 ROIs. All independent factors were modeled as binary indicator variables. Age was split at <65 years and =65 years. For each model, we report the results of the omnibus MANOVA. The results of the univariate tests are reported where the overall MANOVA was significant. While we adopted a multivariate statistical procedure which is designed to help protect against the alpha inflation problem, there may still be the possibility for Type 1 error. Thus we also assessed whether results would survive a Bonferroni correction. Alpha was divided by the number of tested ROIs (i.e. 7) across each imaging modality (i.e. 4), for a corrected alpha of (.05/28) 0.00178. We specify in parentheses which omnibus MANOVA results would survive a Bonferroni correction.

#### 2.4.2 Non-brain analysis

Demographic features, cognitive function, and cardiovascular risk factors including systolic blood pressure, diastolic blood pressure, body mass index, glucose, insulin, total cholesterol and high density lipoprotein were compared between APOE4 carriers and non-carriers, and between participants with and without parental family history of AD using *t*-tests, chi-square, and ANCOVA where appropriate.

## 3 Results

Demographic, cognitive factor scores, and cardiovascular risk factors did not differ between groups based on risk, as shown in Table 1. Of the participants who were APOE4 carriers, 103 also had a parent with AD, while 20 had no family history of AD. Of the participants who were non-carriers, 147 had a parent with AD, while 73 had no parent with AD. Of the APOE4 carriers, 14 participants were homozygous for e4, while the remaining 109 participants were heterozygous. Participants included in the final analysis were all non-demented; one participant had a Mini-Mental State Examination score of 25, six participants had a score of 26, and the remaining participants had MMSE = 27.

### 3.1 Fractional anisotropy

**Fig. 3:**
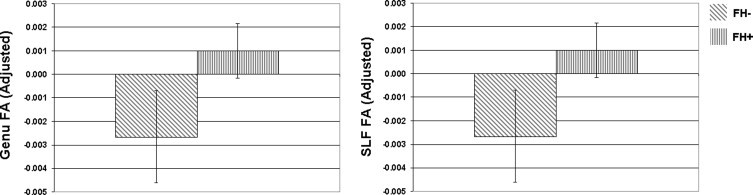
Effects of parental family history on fractional anisotropy. Participants with parental family history (FH) of AD showed higher mean FA in the genu of the corpus callosum and the superior longitudinal fasciculus (SLF) compared to participants who were family history negative. The bar graph shows the regional mean FA adjusted for age and sex. FA = fractional anisotropy; error bars are standard error of the mean. NB: while means in the genu and SLF appear very similar, they were not exactly the same (the correlation between genu and SLF FA across all participants was strong, *R* = .43).

Participants with parental family history of AD differed from those without parental family history, as shown by a significant multivariate effect of FH, Hotelling's *T*(7, 325) = 2.16, *p* < .05. While we expected that participants with positive FH would show lower FA, the univariate tests showed that participants with parental FH of AD had higher FA in the genu, *F*(1,11) = 6.53, *p* = .01 and SLF, *F*(1,11) = 5.07, *p* < .05, compared to participants without parental FH of AD. This effect is shown in Fig. 3.

**Fig. 4:**
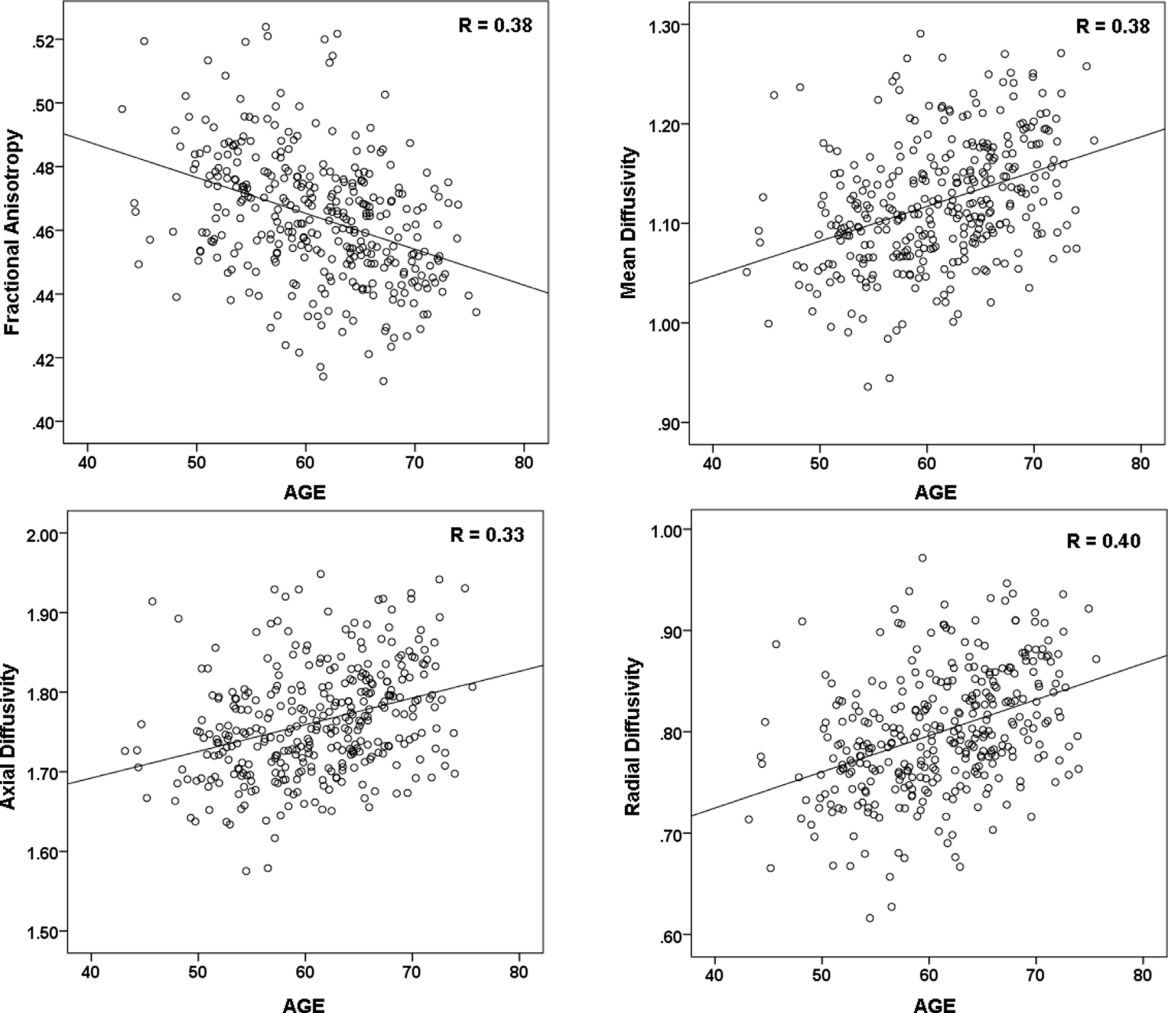
Effect of age. Shown here are the patterns of change in the genu, a region that showed change across all DTI maps. The points represent individual participants. With older age, FA is lower, and mean, axial, and radial diffusivities are higher.

As expected, higher age was associated with lower FA, as shown by a significant multivariate effect of age on FA, Hotelling's *T*(7, 325) = 7.33, *p* < .001 (survives corrected alpha). The univariate tests showed that in the older age group (=65 years) there was lower FA in the genu, *F*(1,11) = 35.17, *p* < .001 (Fig. 4), fornix, *F*(1,11) = 15.61, *p* < .001, and cingulum-CC, *F*(1,11) = 7.82, *p* < .01, compared to the younger age group (<65 years). There was also a significant multivariate effect of sex, Hotelling's *T*(7, 325) = 6.28, *p* < .001 (survives corrected alpha). The univariate tests showed that men had higher FA compared to women in the splenium *F*(1,11) = 10.82, *p* = .001, cingulum-CC, *F*(1,11) = 15.61, *p* < .001, cingulum-HC, *F*(1,11) = 6.71, *p* = .01, SLF, *F*(1,11) = 9.55, *p* = .01 and uncinate, *F*(1,11) = 16.68, *p* = .01.

### 3.2 Mean diffusivity

**Fig. 5:**
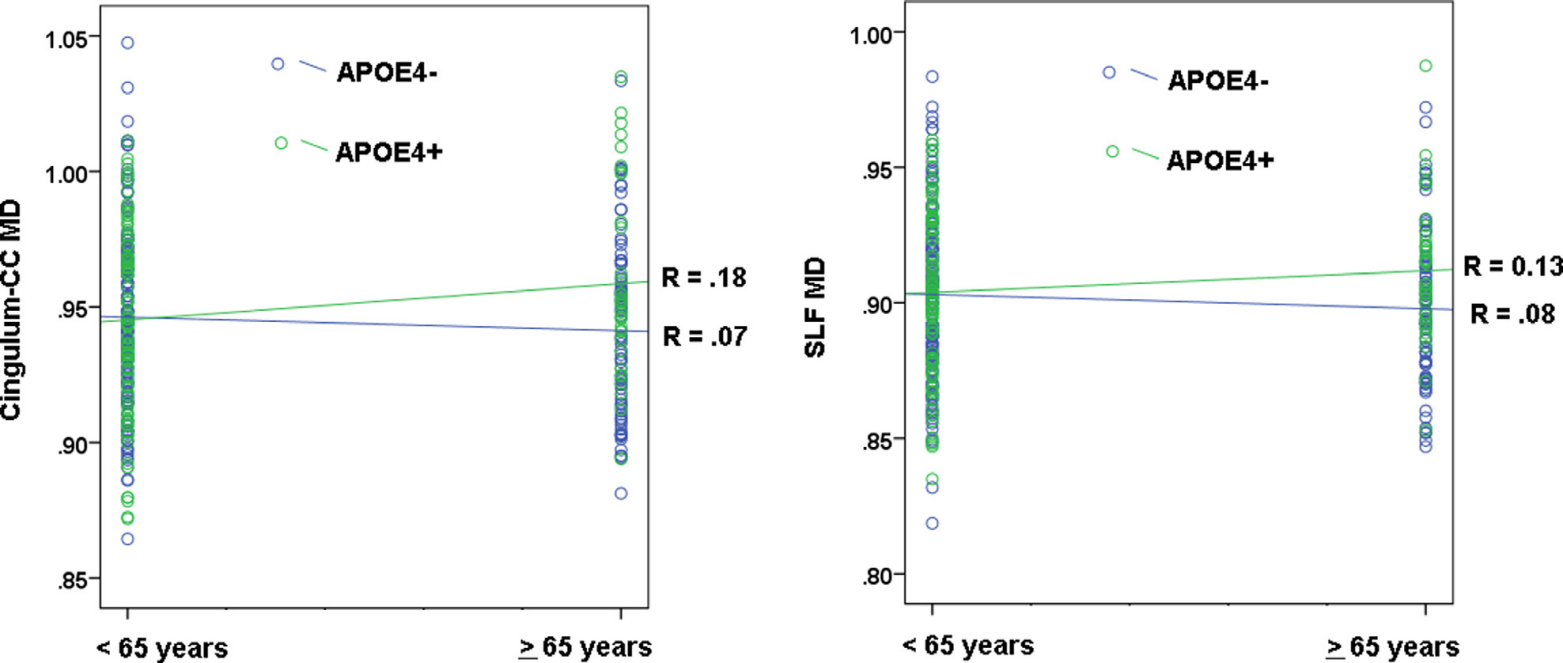
Interaction between APOE4 and age: mean diffusivity. There was a significant interaction between APOE4 and age, whereby carriers of the APOE4 allele showed higher mean diffusivity (MD) in older age (=65 years) compared to younger age (<65 years). The effect was significant in the cingulum bundle adjacent to the cingulate cortex (cingulum-CC), shown on the left, and the superior longitudinal fasciculus (SLF), shown on the right. Participants who were carriers of the APOE4 allele are represented by green circles.

The MANOVA showed no significant main effect of FH or APOE4. There was however, a significant interaction between age and APOE4 genotype, Hotelling's *T*(7, 325) = 2.09, *p* < .05, with the univariate tests indicating that the interaction was present in the cingulum-CC, *F*(1,11) = 11.45, *p* = .001, and in the SLF, *F*(1,11) = 9.01, *p* < .01. As shown in Fig. 5, in older age, APOE4 carriers show higher MD in the cingulum-CC (*M* = 0.96, SD = 0.03) compared to APOE4 non-carriers (*M* = 0.94, SD = 0.03), *F*(1,113) = 6.96, *p* = 0.01, whereas in younger age, there is no significant difference in means, *p* = 0.83. Similarly, in the SLF, older APOE4 carriers show higher MD (*M* = 0.91, SD = 0.03) compared to APOE4 non-carriers (*M* = 0.90, SD = 0.03), *F*(1,113) = 6.54, *p* < 0.05, whereas in younger age, there is no significant difference in means, *p* = 0.85 (Fig. 5).

The MANOVA also showed a significant main effect of age on MD, Hotelling's *T*(7, 325) = 5.75, *p* < .001 (survives corrected alpha). The univariate tests showed that the older age group (=65 years) had higher MD in the genu, *F*(1,11) = 23.06, *p* < .001 (Fig. 4), the splenium, *F*(1,11) = 9.88, *p* < .01, and the fornix, *F*(1,11) = 24.93, *p* < .001. There was no effect of sex on MD.

### 3.3 Axial diffusivity

**Fig. 6:**
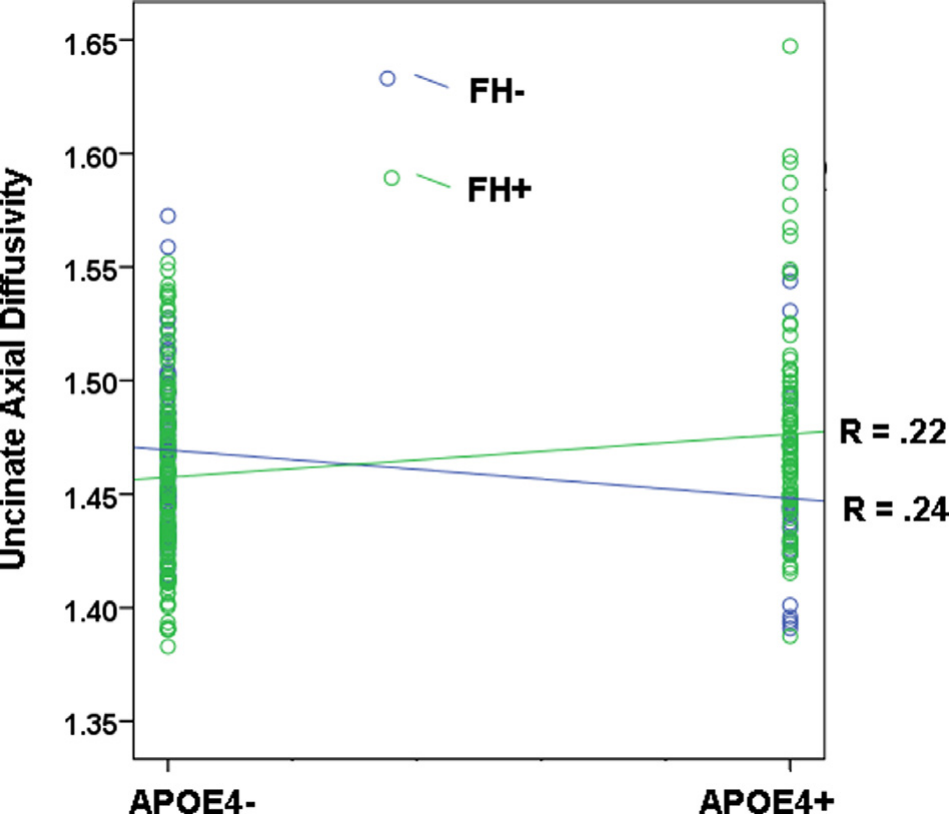
Interaction between APOE4 and parental family history of AD. When evaluating the effect of risk for AD on axial diffusivity, we observed a cross-over interaction. Participants who were both APOE4 positive (APOE4+) and family history positive (FH+) showed higher axial diffusivity in the uncinate fasciculus. In contrast participants who were only FH+ showed lower axial diffusivity compared to FH-. The finding suggests that stages of white matter pathology may differ at different levels of risk for AD. Participants with positive family history of AD are represented by green circles.

The MANOVA demonstrated a significant multivariate effect of FH, Hotelling's *T*(7, 325) = 2.36, *p* < .05, in the uncinate *F*(1,11) = 6.94, *p* < .01. This was explained by a significant interaction between FH and APOE4, Hotelling's *T*(7, 325) = 3.74, *p* = .001, with the univariate analysis indicating that the interaction was significant only in the uncinate, *F*(1,11) = 17.24, *p* < .001. As shown in Fig. 6, the interaction was such that in the APOE4- group, participants who were FH+ had lower axial diffusivity (*M* = 1.46, SD = 0.04) compared to FH- (*M* = 1.47, SD = 0.03), *F*(1,215) = 4.61, *p* < .05; whereas in the APOE4+ group, participants who were FH+ had higher axial diffusivity (*M* = 1.48, SD = 0.04) compared to FH- (*M* = 1.45, SD = 0.04), *F*(1,215) = 9.21, *p* < .01.

The MANOVA also showed a significant main effect of age on axial diffusivity, Hotelling's *T*(7, 325) = 5.02, *p* < .001 (survives corrected alpha). The univariate tests showed that with older age, there was higher axial diffusivity in the genu (Fig. 4), *F*(1,11) = 28.39, *p* < .001, in addition to the splenium, *F*(1,11) = 16.20, *p* < .001, and cingulum-HC, *F*(1,11) = 5.94, *p* < .05. There was no effect of sex on axial diffusivity.

### 3.4 Radial diffusivity

**Fig. 7:**
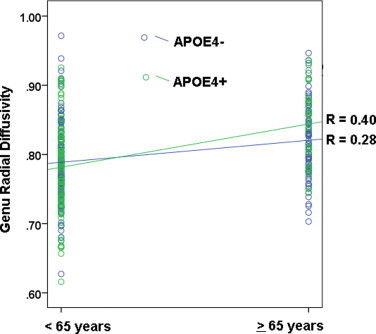
Interaction between APOE4 and age: radial diffusivity. We observed an interaction between APOE4 and age in the genu, where older APOE4 carriers had higher radial diffusivity in the genu compared to APOE4 non-carriers.

The multivariate analysis revealed a significant effect of FH, Hotelling's *T*(7, 325) = 2.97, *p* < .01. However, in follow-up univariate analyses, none of the individual ROIs were statistically significant at the .05 alpha threshold. This suggests that the multivariate finding was not driven by any individual ROI but rather reflects the pooled effect of FH on all ROIs taken together. There were no main effects of APOE4, however, the MANOVA showed a significant interaction between age and APOE4, Hotelling's *T*(7, 325) = 3.52, *p* = .001 (survives corrected alpha), with the univariate tests showing that the interaction was significant in the genu, *F*(1,11) = 8.38, *p* < .01. A plot of the data indicates that older APOE4 carriers showed higher radial diffusivity compared to older non-carriers (Fig. 7). The simple effects test indicated that among younger participants, there was no difference between APOE4 carriers and non-carriers (*p* = .37), whereas among older participants, APOE4 carriers had higher radial diffusivity in the genu (*M* = 0.84, SD = 0.05) compared to APOE4 non-carriers (*M* = 0.82, SD = 0.05), *t*(1, 113) = -2.16, *p* < .05, (shown in Fig. 7).

As found within the analyses of the other DTI indices, results from the MANOVA demonstrated a significant multivariate effect of age, Hotelling's *T*(7, 325) = 7.40, *p* < .001 (survives corrected alpha), with the univariate tests showing that the older age group had higher radial diffusivity in all ROIs studied: the genu (Fig. 4), *F*(1,11) = 38.697, *p* < .001 (as noted above, this region showed a significant interaction between age and APOE4, thus main effects should not be interpreted here), the splenium, *F*(1,11) = 11.60, *p* = .001, the fornix, *F*(1,11) = 25.54, *p* < .001, the cingulum-CC, *F*(1,11) = 14.53, *p* < .001, the cingulum-HC, *F*(1,11) = 19.65, *p* < .001, the SLF, *F*(1,11) = 9.43, *p* < .01 and the uncinate, *F*(1,11) = 3.89, *p* < .05. There was also a multivariate effect of sex, Hotelling's *T*(7, 325) = 4.13, *p* < .001 (survives corrected alpha), with the univariate tests showing that women had higher radial diffusivity compared to men, in the fornix, *F*(1,11) = 5.00, *p* < .05, in the cingulum-CC, *F*(1,11) = 14.01, *p* < .001, and in the SLF, *F*(1,11) = 5.86, *p* < .05.

## 4 Discussion

### 4.1 General

This study tested the effect of age, sex, and risk factors for AD on white matter microstructure in late middle-aged adults. In addition to examining the fairly commonly reported measures of FA and MD, the study also examined effects on axial and radial diffusivities. There was a significant effect of parental family history on FA, in addition to an interaction between family history and APOE4 found in the analysis of axial diffusivity. APOE4 by itself did not have an effect on any of the diffusivity measures, but APOE4 did interact with age. Interactions were found in both the analysis of MD and the analysis of radial diffusivity. Across all participants, age had a significant effect on all tested DTI indices. Sex had an effect on FA and radial diffusivity. In the next sections, we discuss the regional findings in greater detail.

### 4.2 Effect of parental family history

Several studies from our group and others have suggested that family history of AD is associated with preclinical brain changes (Bassett et al., 2006; Fleisher et al., 2009; Johnson et al., 2006, 2007; Schmitz et al., 2004; Trivedi et al., 2007; Xiong et al., 2011; Xu et al., 2009a). Based on prior findings with regard to risk, and patterns observed in AD and mild cognitive impairment, we expected that parental family history of AD would be associated with lower FA, and higher diffusivities. Interestingly, we found that parental family history was associated with higher FA in the genu and the SLF. This was unexpected, given that in AD, the genu undergoes a decline in volume (Black et al., 2000; Canu et al., 2009; Chaim et al., 2007; Di Paola et al., 2010b) and patients show lower FA (Di Paola et al., 2010a; Pitel et al., 2010; Ukmar et al., 2008). While unexpected, there are two possible interpretations that may be considered here. The first is that parental FH of AD has a paradoxical beneficial effect on white matter health. This is unlikely given that parental FH confers an approximate six-fold increased risk for AD (Green et al., 2002; Jayadev et al., 2008; Mayeux et al., 1991) and AD is associated with axonal and myelin degeneration. A second possibility is that FH confers a negative effect on the genu and SLF that is manifesting as increased FA. Loss of fibers, such as occurs in aging and disease could potentially alter FA measurements. The genu of the corpus callosum caries fibers that connect prefrontal cortices, regions that are susceptible to both age and disease related decline. In rhesus macaque, frontal white matter undergoes a 20% reduction in the number of nerve fibers, but degenerative processes are accompanied with continued myelination (Bowley et al., 2010). Electron micrographs in rhesus macaque show loss of axons with preserved myelin sheaths in aged animals (Luebke et al., 2010), a microstructural feature that if not masked by signal related to significant tissue loss, could plausibly result in higher FA. Higher FA in participants with parental family history of AD, controlling for other factors such as age, may reflect axonal loss, and/or reparative myelination. Histological data would provide more conclusive evidence.

Interestingly, a recent study on presymptomatic and symptomatic carriers of the presenilin 1 mutation that results in familial AD indicates that patients in the earliest disease stage (asymptomatic) have higher regional FA compared to healthy controls (Ryan et al., 2013). Significant effects on FA were found in the bilateral thalamus and caudate. Presymptomatic carriers also showed decreased mean diffusivity and axial diffusivity in the right cingulum. In contrast, symptomatic carriers showed patterns typical to those reported in studies of late-onset AD (Acosta-Cabronero et al., 2010; Bozzali et al., 2012), including lower FA, and higher mean, axial, and radial diffusivities across several white matter regions, compared to healthy controls. The exception to this was higher FA observed in the putamen, in symptomatic carriers. Ryan et al. attribute their finding of higher FA to selective degeneration of fibers. For example, degeneration of axonal fibers could manifest as higher FA if the myelin sheath is preserved, or alternatively, fiber loss could contribute to a decline in crossing fibers, which tends to lower measures of anisotropy. At least one prior study found higher diffusion anisotropy in the SLF in patients with mild cognitive impairment compared to control-likely attributable to loss of crossing fibers (Douaud et al., 2011).

In the current study we found higher FA not only in the SLF which is similar to Douaud et al. (2011), but also in the corpus callosum, which contains few crossing fibers. This may suggest that axonal loss is a more likely explanation of higher FA in our presymptomatic cohort. When examining axial diffusivity in our sample, a measure that is suggested to be sensitive to axonal health, we found an interaction between parental family history of AD and APOE4 in the uncinate fasciculus. The interaction was such that in the APOE4 negative group, participants who had parental family history of AD had lower axial diffusivity, whereas in the APOE4 positive group, participants who were also family history positive had higher axial diffusivity. When examined in AD patients, the uncinate shows lower FA (Stricker et al., 2009; Taoka et al., 2006; Yasmin et al., 2008), in addition to higher axial diffusivity compared to controls (Zhang et al., 2009). The data may suggest that in middle-aged adult children of Alzheimer's patients, having family history risk results in early stage axonal pathology that manifests as reduced axial diffusion, while adding the additional risk factor of APOE4 carriage advances the pathology to overt tissue loss, manifesting as higher axial diffusion. The results reported in Ryan et al. (2013), combined with the observations in this study, suggest that different disease stages in AD are accompanied by microstructural alterations that have a non-linear course as the disease progresses. More work will be needed to fully characterize the progression of white matter alteration from preclinical to symptomatic late-onset AD.

### 4.3 Effect of APOE4

The APOE4 gene is the most well-known risk factor for AD. The e4 allele frequency is approximately 15% in the general population but approximately 40% in patients with AD (Roses, 1996). Over the total sample studied here, 36.7% of the participants were carriers of at least one copy of the APOE4 allele. This is likely due to the fact that greater than 2/3 of the sample has a parent with AD. The frequency of APOE4 in the family history positive group was 39.6% compared to 29% in the family history negative group. Previous studies have shown an effect of APOE4 on white matter microstructure (Honea et al., 2009; Ryan et al., 2011; Salminen et al., 2013; Westlye et al., 2012). While we did not find main effects of APOE4, we found an interaction between APOE4 and parental family history of AD as described above, in addition to an interaction between APOE4 and age. Participants in the older age group (=65 years) who were APOE4 carriers showed higher MD compared to non-carriers in the SLF and in the part of the cingulum bundle running adjacent to the cingulate cortex. An interaction between age and APOE was also observed in radial diffusivity, whereby older APOE4 carriers showed higher MD in the genu compared to non-carriers. Interactions between APOE4 and age have been observed previously in studies of white matter (Ryan et al., 2011), cognition (Jochemsen et al., 2012), blood oxygenation level-dependent activation (Nichols et al., 2012), and amyloid deposition measured by Florbetapir positron emission tomography (Fleisher et al., 2013).

Regionally, the observed interactions between APOE4 and age found for MD did not overlap with the regions found for radial diffusivity. The radial diffusivity effect was found in the genu, a region affected both in AD (Di Paola et al., 2010a; Pitel et al., 2010; Ukmar et al., 2008) and in normal aging (Bucur et al., 2008; Madden et al., 2009; Zahr et al., 2009). The interaction between APOE4 and age on MD was found in the SLF, which carries fibers that connect wide-spread parts of the cerebrum, including the frontal, occipital, parietal, and temporal lobes (Makris et al., 2005). SLF fibers are affected in AD (Douaud et al., 2011; Guo et al., 2012; Pievani et al., 2010; Rose et al., 2000; Stricker et al., 2009) and in mild cognitive impairment (Amlien et al., 2013; Liu et al., 2011). The MD effect was also observed in the cingulum bundle, which carries fibers connecting association cortices in the frontal, parietal, and temporal lobes, thalamus, insular cortices, and the hippocampal formation, (Goldman-Rakic et al., 1984; Makris et al., 2002), and fibers originating from the cingulate cortex (Mufson and Pandya, 1984). The posterior cingulate gyrus is affected in early stages of AD and this cortical region shows alterations in people at increased risk for AD, including APOE4 carriers (Reiman et al., 1996; Valla et al., 2010) and in adults with parental family history of AD (Johnson et al., 2007; Mosconi et al., 2007; Scarmeas et al., 2004; Small et al., 2000). Similarly, the cingulum bundle, which is rich in fibers whose cell bodies are located in the posterior cingulate cortex, is likely one of the earliest white matter tracts affected by AD (Villain et al., 2008, 2010). Adults who are at increased risk for AD show differences in the cingulum, including carriers of APOE4 (Smith et al., 2010) and those with parental family history of AD (Bendlin et al., 2010), reinforcing the notion that this region is disrupted early in the disease process. In a prior study from our group (Bendlin et al., 2010), we found a main effect of parental family history of AD in the posterior portion of the cingulum bundle, adjacent to the posterior cingulate cortex, in addition to an additive effect of APOE4. The primary differences in the current study include a larger sample of adults, with a slightly higher mean age, acquisition of higher resolution data, parallel imaging, a better characterized diffusion tensor, newer developments in image registration, and an ROI approach. Overall, both studies point toward an early vulnerability of the cingulum bundle.

### 4.4 Effects of age and sex

We found an effect of age on all of the DTI maps, and it is worth noting that the effects of age appeared to be more robust than the effects of AD risk factors given that all of the age results survived correction for multiple comparisons. The effect was consistent across indices in the genu of the corpus callosum, a region that has been shown to change with age in several prior DTI studies (Abe et al., 2002; Bucur et al., 2008; Davis et al., 2009; Madden et al., 2004; Pfefferbaum et al., 2000; Salat et al., 2005). With some differences in which regions differed across DTI maps, age group also had a significant effect on the splenium, the fornix, the portion of the cingulum bundle running adjacent to the cingulate cortex, and the portion projecting to the hippocampus. The pattern of age related change was consistent with other studies, with FA showing a decrease in older age, and MD and radial diffusivity increasing. Axial diffusivity has been reported to either decrease or increase with age, depending on the region studied (Bennett et al., 2010). In this study, axial diffusivity in the genu, the splenium, and the portion of the cingulum projecting to the hippocampus was higher in the older age group, which has been observed in other studies of aging (Sullivan et al., 2010). It should be noted that the effects of age may be overestimated, given that certain structures such as the genu and the fornix are susceptible to partial volume effects due to proximal CSF (Metzler-Baddeley et al., 2012). One approach to minimizing CSF contamination is to fit multi-tissue compartment models to the diffusion weighted MR signal (Alexander et al., 2001a), which may be best achieved by obtaining multiple shell diffusion weighted data which allows more accurate fitting of a multiple-component model (Zhang et al., 2012). Features of our study which reduced CSF contamination to a certain extent include thresholding of the region of interest masks using an FA cut-off of 0.2 to reduce gray matter and CSF inclusion, and a reasonable voxel size of 2.5 mm^3^. In this sample of participants, neither the uncinate nor the SLF showed a group effect of age, although this may have been due to the age split at 65 years for the MANOVA models.

With regard to sex, differences were only observed in FA and radial diffusivity. Both of these effects survived correction for multiple comparisons, whereas the effect of family history on FA did not survive correction for multiple comparisons. Women showed lower FA in the splenium, both portions of the cingulum, the SLF, and the uncinate. Higher radial diffusivity in women was observed in the cingulum adjacent to the cingulate cortex, in the fornix, and in the SLF. Total and regional white matter volume is higher in men compared to women (Driesen, 1995; Luders et al., 2002; Salat et al., 2009) which may result in less partial volume effect on diffusion measures in men; although some regional microstructural differences between men and women may also be present (Westerhausen et al., 2011). We did not find that sex interacted with any of the AD risk variables, but the results underscore that it is important to control for sex in DTI studies due to potential sex differences on the measures.

### 4.5 Limitations

A few limitations deserve mention. While the sample is large, this was a cross-sectional analysis. Individual change over time is likely to be a better metric for assessing risk of pathological aging compared to measurements at one time point. Further, the sample was heavily weighted toward female participants and participants with risk; approximately 67% of the participants were women and less than 20% of the participants had neither APOE4 nor family history of AD. We should also note that APOE4 is a single genetic risk, and parental family history of AD likely encompasses several genetic risk factors. More work is needed to understand the contributions of other AD-risk genes on white matter microstructure. Lastly, in the absence of histology or more direct MRI proxy measures of myelin and axonal density, the interpretation of differences in measures such as FA and the lambda maps remains speculative. Advances in multi-shell diffusion-weighted imaging (Hsu et al., 2008), application of novel models (Zhang et al., 2012), and acquisition of complementary imaging techniques (Deoni et al., 2008) are expected to further clarify the white matter changes occurring in age and disease.

### 4.6 Summary

Both APOE4 and parental family history of AD have an effect on white matter microstructure in cognitively healthy late middle-aged adults. Overall, the findings in this study suggest that patterns of white matter microstructural change, particularly at various disease stages may be more complex than initially thought. We observed higher FA in participants with greater risk of developing AD due to parental family history, which while unexpected, has been observed in other patient groups that include Williams Syndrome (Hoeft et al., 2007), fibromyalgia (Lutz et al., 2008), bipolar disorder (Yurgelun-Todd et al., 2007) and multiple sclerosis (Hannoun et al., 2012). Additional evidence for a non-linear pattern was apparent in participants with varying risks, where participants who had a parent with AD, but did not carry APOE4 showed lower axial diffusivity in the uncinate fasciculus, whereas participants who had both risk factors for AD had higher axial diffusivity in this region. Similarly, being older and carrying an APOE4 allele were associated with higher mean and radial diffusivities, an observation consistent with more advanced neurodegeneration. Additional work is needed to characterize the course of brain changes that occur at presymptomatic disease stages in late-onset AD.

## Figures and Tables

**Table 1:** Demographics, vascular risk and cognitive function among risk groups.

	APOE4+		APOE4-				FH+		FH-			
Demographics and vascular risk factors	*M*	SD	*M*	SD	*t*	*p*	*M*	SD	*M*	SD	*t*	*p*
Age (years)	60.12	6.81	61.54	6.63	1.89	0.06	60.29	6.89	62.96	5.84	3.32	0.001
Education (years)	16.33	2.22	16.24	2.47	–0.34	0.74	16.33	2.33	16.10	2.50	–0.81	0.42
Systolic BP	122.85	15.41	125.28	15.89	1.38	0.17	125.10	15.51	122.54	16.3	–1.34	0.18
Diastolic BP	73.82	9.1	74.16	9.85	0.72	0.48	74.23	9.53	72.92	9.72	–1.12	0.26
BMI	28.40	5.47	27.84	5.37	–0.92	0.36	28.33	5.31	27.26	5.59	–1.64	0.10
Fasting glucose	93.92	13.58	96.70	20.28	1.30	0.19	96.02	20.1	94.92	11.4	–0.49	0.63
Fasting insulin	8.51	5.36	9.97	8.98	1.62	0.11	9.66	8.22	8.84	6.95	–.84	0.40
Total cholesterol	197.58	32.63	194.32	35.67	–0.83	0.41	196.65	34.59	192.41	34.59	–1.01	0.31
HDL cholesterol	58.34	16.8	61.56	19.08	1.53	0.13	59.58	17.67	62.70	19.98	1.37	0.17
												
	*N*		*N*		?^2^	*p*	*N*		*N*		?^2^	*p*
Sex (female)	86 (68%)		144 (66%)		0.13	0.72	166 (66%)		64 (69%)		0.18	0.67
												
Cognitive factor score	*M*	SD	*M*	SD	*F*	*p*	*M*	SD	*M*	SD	*F*	*p*
Speed and Flexibility (Z)	0.28	0.92	0.15	0.91	0.40	0.53	0.24	0.90	0.09	0.94	0.03	0.86
Working Memory (Z)	0.21	1.00	0.07	1.01	1.36	0.24	0.06	1.01	0.26	0.99	3.07	0.08
Verbal Learning (Z)	0.084	1.03	0.055	1.00	0.03	0.87	0.03	1.00	0.17	1.02	2.75	0.10
Immediate Memory (Z)	–0.04	1.06	0.05	1.02	1.20	0.27	–0.02	1.05	0.10	0.98	1.78	0.18

Cognitive factor scores were derived from a factor analytic study of the WRAP neuropsychological battery. Individual neuropsychological tests loaded onto separate factors. Rey Auditory Verbal Learning Test (Spreen and Strauss, 1998), Trials 1 and 2 loaded onto  the Immediate Memory factor. Rey Auditory Verbal Learning Test (Spreen and Strauss, 1998), Trials 3–5 and Delayed Recall Trial loaded onto the Verbal Learning and Memory factor.  Wechsler Adult Intelligence Scale—3rd edition (Wechsler, 1997), Digit Span, Arithmetic, and Letter-Numbering Sequencing subtests loaded onto the Working Memory factor. The interference trial from the Stroop test (Trenerry, 1989), and Trail Making Test A and B (Reitan and Wolfson, 1993) loaded onto the Speed and Flexibility factor. The Speed and Flexibility factor score was unavailable for nine participants. The ANCOVA for cognitive function controlled for age, years of education, and sex. APOE4: apolipoprotein epsilon 4 allele; FH: family history of Alzheimer's disease; M: mean; SD: standard deviation; BP: blood pressure; BMI: body mass index; HDL: high density lipoprotein. Missing data: A sub-set of participants did not have vascular risk factors available; blood pressure, BMI, and cholesterol were missing for one participant, insulin was missing for 12 participants, and HDL and glucose were missing for 11 participants.
